# Society Meetings

**Published:** 1915-01

**Authors:** 


### SOCIETY MEETINGS

---

Brief reports and announcements of meetings of societies of Western New York, and those of general scope, are requested from Secretaries. Copy should be on hand the fifteenth of the month. Full transactions will be published at cost of composition.

---

**DOCTOR: Is your Society properly represented here? If not, please bring our standing announcement to the attention of the members.**

---

**The Gross Medical Club** held their final meeting of 1914 at the Hotel Touraine, Friday, December 18, as the guest of Dr. D. C. MacKenney; Dr. A. G. Bennett, president, presided.

The paper, "The Workingman's Compensation Law," was presented by Dr. Lawrence H. Smith, of East Aurora (will soon appear in print). This was quite widely discussed by the members present, especially by Dr. James E. King, who complimented the writer for presenting a subject of such magnitude in such a clear and concise manner. Dr. B. E. Smith, of Angola, N. Y., was elected to active membership.

Following the business meeting the ladies were admitted and for over an hour all were most highly entertained by Mrs. Swift, the well known monologist. Dinner followed in the private dining-room, the host presenting each lady and gentleman with a souvenir, many of which were reminders, especially to the bachelors present.

---

**The Medical Society of the County of Chautauqua** held its annual meeting in Jamestown, December 8. Drs. Irving W. Potter, C. F. Goldsborough, Frank E. Brundage and Arthur G. Bennett, of Buffalo, presented papers. Officers were elected as follows: president, Fred C. Rice, of Ripley; vice-presidents, A. W. Dods, of Fredonia; J. H. Kellogg, of Bemus Point; secretary, J. W. Morris, of Jamestown; treasurer, Geo. F. Smith, of Falconer; censor (3 years) Morris N. Bemus, of Jamestown; delegate to New York State Society, V. M. Griswold, of Fredonia; alternate, G. W. Cottis, of Jamestown. Joseph Chilli, of Fredonia; Charles B. Mosher, of Dunkirk, and John W. Henderson, of Stockton were admitted to membership.

---

**Medical Fraternity Meets in Philadelphia.** Two hundred physicians and medical students, attended the national convention of the Nu Sigma Nu Medical Fraternity held in Philadelphia on November 28th. Practically all the medical schools in America were represented by the delegates, who were entertained by two local chapters, Rho Chapter of Jefferson Medical College and Lambda Chapter of the University of Pennsylvania. The highest honor in the fraternity was awarded Dr. J. Playfair McMurrich, professor of anatomy at the University of Toronto, for distinguished work in anatomical research, and will be formally conferred upon him at the next convention, to be held in 1916.

---

**The Medical Society of the County of Monroe** held its annual meeting December 15. The retiring president, Dr. A. C. Snell, gave an address. Under date of November 1, the society has issued the following notice:

To the Legal Profession of this County:

Please observe that our society has again prepared a list of experts in insanity for another year. This list is the same as that of last year, namely: E. B. Angell, Eveline P. Ballintine, Edward L. Hanes, Wallace J. Herriman, Eugene H. Howard, Peter Wyckoff Neefus, Mary C. Nickerson, Sarah G. Pierson, Ezra B. Potter, Alvah C. Remington, Alexander L. Smith, C. A. Van der Beek, Willard H. Veeder, and Irving Lee Walker.

Our society is convinced that attempts at the regulation of medical expert testimony may fail for some time to come, and so it has decided to attack the problem in a new way. The names are first acted upon by our Board of Censors and then voted upon by the society by ballot after due notice, a two-thirds vote being required to elect. In this way our society feels that it can vouch for these experts. It vouches for no others at present. You may, of course, use these experts or not as you wish. However, the inference is that should you use others no blame can come to our profession because of any medico-legal scandal arising therefrom.

Very respectfully, Charles W. Hennington,

Secretary.

---

**The Rochester Medical Association** held its first regular meeting December 2. Dr. Henry L. Elsner of Syracuse gave a paper on the Forecast of So-called Metasyphilitic Diseases of the Nervous System and Dr. Edgar M. McGuire, of Buffalo, on Surgery of the Duodenum.

---

**The Rochester Pathological Society** met, December 3. Dr. W. F. Plumley gave a paper on Venereal Prophylaxis.

---

**The Rochester Academy of Medicine,** Section IV, Public Health, met, December 9. The program was as follows:

Hyperthyroidism ................. Dr. J. R. Culkin
Basedow's Disease ............... Dr. W. V. Ewers
Hypothyroidism and Myxedema .. Dr. C. D. Young
Apparatus for Recording Tremors, Dr. R. M. Moore

---

**The Buffalo Society of Sanitary and Moral Prophylaxis** held a meeting December 4.

---

**The Elmira Academy of Medicine** met, December 2. The president, Dr. J. A. Bennett, delivered an address and Dr. R. G. Loop presented a paper on Non-operative Gynaecology.

---

**The Buffalo Academy of Medicine** has held the following meetings since our previous notice: Pathologic Section, November 25, Recent Studies in the Use of Sera and Vaccines in Treatment and Immunization, Dr. Wm. H. Park, of New York.

Stated meeting, under auspices of Surgical Section, December 2, Methods in Diagnosis and Results, Dr. W. D. Johnson, of Batavia; Present Status of the Goitre Problem, Dr. G. W. Cottis of Jamestown; (both to appear in this Journal).

Medical Section, December 9, Pulmonary Complications of Venous Thrombosis, Dr. Lewis A. Conner of New York;

Section of Obstetrics and Gynaecology, December 16, Caesarian Section for Eclampisia, Dr. Asa B. Davis of New York; Technic of Operation, Dr. Irving W. Potter of Buffalo; Discussion opened by Dr. W. M. Brown, of Rochester.

Section of Pathology, December 23, Dr. Harvey R. Gaylord, of Buffalo, Remarks on Certain Developments in Cancer Research.

---

**American College of Surgeons.** At the third convocation held in Washington, November 16, 1914, the following honorary fellows were admitted: Dudley P. Allen, of Cleveland; Wm. C. Gorgas, of Washington; Lewis S. Pilcher, of Brooklyn; Sir Thomas George Roddick, of Montreal; J. William White, of Philadelphia. 646 fellows were admitted. $250,000 of the million endowment fund had been subscribed. 2700 members had been admitted altogether and about 2,000 applicants remained. Washington was favored as the site of the permanent home of the College. The American Institute of Homoeopathy was added to the 15 societies recognized. The fees were fixed at $25 initiation and $5 annually for 5 years, or $50 for life membership. Beside the requirement of graduation at least five years before admission, personal and professional standing, and specialization in surgical lines, the following tentative plan for the Committee on Examination was proposed:

1. Evidence that applicant has served at least one year as a hospital interne and three years as assistant, or one year as first assistant to a surgeon of recognized ability and with an adequate hospital service. From those who were graduated before 1915 an equivalent surgical experience shall be acceptable, especial importance being attached to laboratory and research work.

2. Evidence that he has visited other surgical clinics and laboratories than those to which he has been officially appointed, giving the dates of such visits, the time spent, and a brief summary of the work witnessed or performed.

3. An abstract of at least fifty consecutive major operations which he has himself performed, this abstract to contain the name and address of the doctor or consultant referring the case; the pre-operative diagnosis; the anaesthetic given, by whom, the quantity and the time of administration; the date of operation, and a brief description of it, with a note of the time required for its performance, calculated from the first incision to the beginning of the application of the dressing; the post-operative course, and a mention of complications, if such occurred; not only those conditions usually classed as such, but consecutive bleeding which calls for measures directed toward its control, haematoma of sufficient extent to require evacuation or drainage or suppuration, as slight as a stitch abscess, are to be regarded as complications. The condition on discharge from the hospital with subsequent course of the case up to the date of application for membership, or as near this as practicable. The applicant shall supplement his individual report of operations by a further abstract report of at least fifty cases in which he has acted as assistant.

4. All applicants for fellowship to the American College of Surgeons whose date of graduation is 1920 or later shall be graduates from medical schools which shall have demanded of its matriculates two years of collegiate training, or the equivalent, including biology, chemistry, and physics. If the candidate's school of graduation be not accredited by the American College of Surgeons, he shall be required to pass a technical examination.

5. Surgeons widely recognized by the profession as leaders of progress and exponents of finished technique, by a unanimous vote of the Board of Regents may be admitted to fellowship on recommendation of the Committee on Examination.

Officers were elected as follows:
President—Dr. J. M. T. Finney.
First Vice-President—Dr. W. W. Chipman.
Second Vice-President—Dr. Rudolph Matas.
General Secretary—Dr. Franklin H. Martin.
Treasurer—Dr. A. J. Ochsner.

---

The 93rd Annual Meeting of the **Medical Society of the County of Erie** was held Dec. 21. President Woodruff presiding.

Treasurer Lytle presented his annual report, subject to some changes before the end of the year.

| | |
|---|---|
| Total receipts during the year | $3,637.01 |
| Paid to Med. Soc. S. of N. Y. for membership dues | 1,602.00 |
| After deducting other expenses leaves balance in treasury of | $1,170.82 |

The following were elected to membership: Louis J. Regan, 949 William St.; Edw. B. Bangasser, 484 Leroy Ave.; Leonard Duszynski, 1073 Broadway; S. E. Inda, 947 Sycamore St.; Elmer H. Stumpf, 201 Linwood Ave.; J. Stewart Banta, 407 Perry St.; Grace M. Elmendorf, 649 Spring St.; Peter J. Barone, 294 Cedar St.; Jerome A. Murphy, 505 Genesee St.; Frank F. Roney, 393 Winslow Ave.; Raymond Hensel, 151 West Utica St.; Jos. A. Nowicki, 830 Fillmore Ave.; J. Grafton Jones, 473 Virginia St.; Edw. H. Meister, 90 High St.; Jasper D. Wooster, Wales Center, N. Y.; John D. O'Brien, 160 Alabama St.; Edith M. Lehnis, 1444 Broadway; James L. Mangano, 95 Front Ave.; Francis Argus, 770 East Ferry St.; John J. Kohlhas, 802 Genesee St.

In connection with his list of applicants, Chairman C. W. Wende presented the following annual report:

"The membership of this society, at the close of our last annual meeting, consisted of 524 members, of which 523 were active and 1 honorary; of this number three have died and one has removed during the past year, leaving a total of 520 members. To this may be added for active members 125, of whom four have applied for retirement and, if elected, will leave a total membership of 641.

Dr. Henry R. Hopkins, Chairman of the Committee on Public Health, presented a verbal report, and moved the following resolution which was adopted:

"Whereas, the Medical Society of the County of Erie, State of New York, at its annual meeting of December 18th, 1911, adopted resolutions requesting those in authority when reporting cases of small-pox to report the facts of vaccination or non-vaccination of said cases, and

Whereas, subsequent to that date—December 18th, 1911, nine of our States have so reported their cases of small-pox, and

Whereas, the United States Public Health Reports for October 2d, 1914, give such reports, and

Whereas, these reports mention 20,835 cases of small-pox, only 540 of which cases have been vaccinated within seven years preceding the attack, and

Whereas, we believe the same immunity towards small-pox is enjoyed by all recently and properly vaccinated persons, and

Whereas, we also believe that the national expression of such immunity would constitute a safeguard of vast proportions, therefore.

Resolved, that the Medical Society of the County of Erie earnestly requests those in authority—the Medical Society of the State of New York, The American Medical Association, and the United States Public Health Service—to continue their interest and support of this mode of reporting cases of small-pox until the same shall be the rule in every State and Territory of America.

Resolved, that copies of these resolutions be sent, under the seal of this society, to such officers and associations as have been or may be interested in this important item of preventive medicine.

Joseph P. Gimbrone, M. D., J. F. Whitwell, Henry Reed Hopkins, Chairman, Committee on Public Health.''

Dr. John D. Bonnar, Chairman of the Board of Censors, submitted a very interesting report, with a report of the Counsel of the Society for the past year.

On motion of Dr. Bennett, a resolution was adopted instructing Dr. Albert T. Lytle, Chairman of the Committee of Arrangements for the coming meeting of the Medical Society of the State of New York, to secure the valuable exhibit of the American Medical Association.

On motion of Dr. Bonnar, the usual honorarium of $100.00 was voted to be given to Mr. Albert L. Harrison, attorney of the Board of Censors for his services during the past year.

Officers for 1915:

President—Dr. Arthur W. Burd.

First Vice-president—Dr. Franklin W. Barrows.

Second Vice-president—Dr. Irving W. Potter.

Secretary—Dr. Franklin C. Gram.

Treasurer—Dr. Albert T. Lytle.

Censors—Drs. John D. Bonnar, Francis E. Fronczak, Arthur G. Bennett, Archibald D. Carpenter, Charles Battaglia.

Chairman Com. on Legislation—Dr. Harvey R. Gaylord.

Chairman Com. on Public Health—Dr. Henry R. Hopkins.

Chairman Com. on Membership—Dr. William F. Jacobs.

Chairman Com. on Education—Dr. John V. Woodruff.

Delegates to State Society—Drs. Charles G. Stockton,

Thomas H. McKee, Arthur W. Hurd, Edward L. Frost, Edith R. Hatch.

President Whitwell then called President-elect Hurd to the chair, and read his annual report as retiring president, in which he laid strong emphasis on the economic problems confronting the physicians of the present and the possible effects on the future.

A letter, from Dr. Grover W. Wende, President of the Medical Society of the State of New York, was read by the Secretary, in which he directed attention to pernicious legislation secured in the past and calling special attention to the obnoxious bills which were vetoed by the Governor last year, after having passed the Senate and Assembly, and which are, again, likely to be introduced at the coming session of the State Legislature.

Dr. Lytle moved that the Secretary be empowered to send, to each member of the society, a communication, embodying the facts contained in State President Wende's letter, also to include the list of Senators and Assemblymen, and requesting the members to use their personal influence on these legislators, but that this should be done in co-operation with the incoming Committee on Legislation. Carried.

The chair called upon Dr. Wende to supplement his letter with personal remarks.

On motion of Dr. Bennett, the usual honorarium of $100.00 each was voted to Secretary, Treasurer and Chairman of the Board of Censors for services during the past year.

